# PAP Polypeptide Promotes Osteogenesis in Jaw Bone Defect Repair by Inhibiting Inflammatory Reactions

**DOI:** 10.3389/fbioe.2022.916330

**Published:** 2022-06-02

**Authors:** Ke Guo, Haoming Zhao, Guokun Chen, Ying Zhang, Yu Wang, Liang Huo, Shoufu Sun, Wenjia Wei

**Affiliations:** ^1^ Department of Stomatology, Tongren Hospital, Shanghai Jiao Tong University School of Medicine, Shanghai, China; ^2^ Shandong Key Laboratory of Oral Tissue Regeneration and Shandong Engineering Laboratory for Dental Materials and Oral Tissue Regeneration, Department of Oral and Maxillofacial Surgery, School and Hospital of Stomatology, Cheeloo College of Medicine, Shandong University, Jinan, China; ^3^ Nursing Department, Shanghai Ninth People’s Hospital, Shanghai Jiao Tong University School of Medicine, Shanghai, China; ^4^ Department of Oral Surgery, Shanghai Ninth People’s Hospital, Shanghai Jiao Tong University School of Medicine, Shanghai, China

**Keywords:** polypeptide, anti-inflammatory, macrophage, polarization, BMSC, Jaw defect

## Abstract

Jaw defects are common in oral and maxillofacial diseases and require surgical repair in extreme cases. Given the limitations in availability and efficacy of autologous bone grafts or allografts, great effort has been made in finding suitable, biocompatible, and effective artificial bone materials. Considering the key role of inflammation in bone resorption, we sought to identify a polypeptide with anti-inflammatory and bone-promoting effects. Rat bone marrow-derived mesenchymal cells (BMSCs) were treated with lipopolysaccharide (LPS) to induce an inflammatory environment, and 1,538 differentially abundant polypeptides were identified using mass spectrometry. Based on mass spectrometry signal intensity, multiple of difference, and structural stability, PAP was screened out as the polypeptide with the lowest abundance in the inflammatory condition. PAP showed no cytotoxicity to BMSCs with increasing concentrations. PAP (10 μM) also increased alkaline phosphatase activity and mRNA expression of *Ocn*, *Bmp2*, and *Runx2* in a concentration-dependent manner, which confirmed that it can promote osteogenic induction of rat BMSCs. Moreover, PAP reduced LPS-induced expression of inflammatory cytokines (TNF-α, IL-1β, IL-6) and reactive oxygen species and inhibited polarization of RAW 264.7 macrophages to the inflammatory type. Finally, a skull defect mouse model was established, and mice were injected with LPS and/or PAP. Micro-CT, histological analysis, and immunohistochemical staining showed that PAP significantly reduced the number of LPS-induced bone resorption pits and maintained bone integrity. Overall, the polypeptide PAP screened using LPS stimulation of BMSCs is not cytotoxic and can inhibit the inflammatory reaction process to promote osteogenesis. This study thus provides a basis for development of PAP as a new osteogenic material in the repair of jaw defects.

## 1 Introduction

Bone defects caused by aging, trauma, infection, tumor, surgical debridement of osteomyelitis, and various congenital diseases (Götz et al., 2015) are the most common forms of oral and maxillofacial disease. The jaw is one of the most important structures in the maxillofacial region, which plays a role in maintaining maxillofacial morphology and enabling occlusal and masticatory ability. Consequently, jaw defects affect the physiological functions of mastication, swallowing, and breathing, which significantly reduce quality of life and can even be life-threatening in severe cases. Therefore, effective mandibular prosthesis is needed to reconstruct and regenerate the mandibular bone.

Currently, repair and treatment of jaw defects are limited to autologous bone grafts, allografts, and artificial bone grafts. Autogenous bone grafting is the gold standard for bone defect repair owing to its many advantages, including good biocompatibility and strong osteogenic ability, compared with those of non-autogenous bone grafting materials. The clinical success rate of autologous bone transplantation can reach up to 60–95%. However, additional surgeries are needed to remove the bone, which can lead to problems such as donor site injury, high treatment cost, postoperative hematoma, and increased risk of infection. More importantly, the source and quantity of the autogenous material are limited. Compared with autologous bone transplantation, allogeneic bone transplantation has many advantages such as convenience, a relatively abundant source, inactivation of bone cells after treatment, low immunogenicity, and structural support (Amini et al., 2012; Pilipchuk et al., 2015). Freeze-drying, irradiation, or chemical treatment are commonly used in clinical practice to reduce immunogenicity. Artificial bone materials commonly used in clinical practice are often available in the form of bone powder, produced by pulverizing animal bones into powder and then removing antigens (Griffith, 2002; Jung et al., 2015). However, these allografts still have limitations such as low induction activity, poor quality of fused bone, high absorption rate, frequent immune rejection, inflammation, and other deficiencies, affecting the clinical efficacy of existing bone grafting methods and resulting in a low complete bone union rate (Tischler, 2002).

Accumulating evidence demonstrates that the inflammatory response is the most important local factor affecting the success rate of bone grafting and bone healing rate. As a defense mechanism, an appropriate inflammatory response is conducive to bone healing. However, long-term chronic inflammation caused by infection or other factors has a significant inhibitory effect on the bone healing process (Koh et al., 2011; Guo et al., 2018; Guo et al., 2019; Ke et al., 2019; Guo et al., 2020). Therefore, a material that can suppress the inflammatory environment would be ideal for bone grafting in jaw repair.

Under physiological conditions, bone marrow-derived mesenchymal stem cells (BMSCs) are capable of self-renewal and repair owing to the regulation of intracellular gene signals and extracellular signals in the cell microenvironment. During osteogenesis, BMSCs synthesize bone morphogenic protein (BMP)-2, alkaline phosphatase (ALP), osteopontin, and collagen I through the BMP/SMADs and Wnt/β-catenin pathways. RUNX2, as a key transcription factor in the osteogenesis of BMSCs, regulates the expression of these proteins. However, in an inflammatory environment, these traditional signaling pathways are blocked, resulting in impaired cellular activity and inability to repair tissue damage. BMSCs are often subjected to ischemia, hypoxia, and inflammation in the inflammatory microenvironment, which has a significant impact on the self-renewal ability and multi-directional differentiation potential of the cells, leading to decline of the osteogenic differentiation ability and inhibition of the bone formation ability of osteoblasts (Hah et al., 2011). In addition, recent studies have confirmed that pro-inflammatory cytokines in the inflammatory environment also have significant effects on the function of osteoblasts by 1) directly inhibiting the differentiation, maturation, and osteogenesis of osteoblasts; 2) stimulating osteoblasts to secrete osteoclast regulatory factors to promote bone resorption; 3) inducing osteoblast apoptosis and reducing the number of osteoblasts to inhibit bone formation; and 4) influencing the regulatory effects of other pro-differentiation cytokines or growth factors on osteoblasts (Holt et al., 2007; Purdue et al., 2007; Ma et al., 2013). During osteoclysis, bone marrow macrophages (BMMs) synthesize TRAP, CTSK, and other osteoclast-related proteins through the RANKL pathway, which are regulated by the key transcription factors C-Fos and NFATc1. Thus, the inflammatory reaction has a clear influence on the osteoclast process. BMMs recruit cells for phagocytosis of foreign bodies during the inflammatory response and induce the release of chemokines and pro-inflammatory cytokines such as tumor necrosis factor (TNF)-α, interleukin (IL)-6, IL-11, and prostaglandin E2 (Mori et al., 2009; Sokos et al., 2015). The presence of local inflammatory factors can further induce and stimulate the differentiation of BMMs and signal pathways related to bone resorption function; synthesize TRAP, CTSK, c-Fos, and NFATc1; and induce the continuous process of bone resorption (Aloya et al., 2006; Pountos et al., 2016). Therefore, it is particularly important to inhibit the osteoclast differentiation of BMMs and promote the osteogenic differentiation of BMSCs in the inflammatory microenvironment.

The potential to repair bone defects with scaffolding materials and polypeptide complexes has attracted substantial research attention (Gabet et al., 2004). Polypeptides not only inhibit inflammation and overcome the limitations of conventional bone repair materials but also may generate bone tissue with good biological activity. In recent years, advances in polypeptide omics have helped identify small peptides with low molecular weight and easy entry into cells, along with metabolites with non-toxic side effects that can be secreted from cells that play important roles in bone formation processes (Bab et al., 1992). Some of these peptides have shown anti-inflammatory, supportive, bone-stimulating, and bone healing properties. For example, the peptide H-RN was shown to inhibit inflammatory responses both *in vitro* and *in vivo* through the PI3K/AKT/IKK/NF-κB signaling pathway, thus exhibiting osteogenic potential (Wang et al., 2014). RGD peptide was shown to promote the adhesion or osteogenic differentiation of human MSCs on a variety of bioactive materials (Palumbo et al., 2019). Yang et al. (2018) demonstrated that BFP-1 can enhance the expression and activity of osteogenic genes, proteins, transcription factors, and enzymes, thus promoting the osteogenic differentiation of BMSCs. Moreover, multiple BMP-2–derived peptides such as OP peptide, BMP-2 residue 73–92, and BMP-2 residue 32-48 have been shown to enhance expression of the osteogenic differentiation marker ALP, *RUNX2*, and related proteins of MSCs. Overall, these results indicate that BMP-2–derived peptides can promote osteogenic differentiation (Zhou et al., 2015; Zhang et al., 2016; Lee et al., 2017).

To further improve the ability of osteogenic materials in jaw bone repair in an inflammatory environment, in this study, we screened out target peptides in lipopolysaccharide (LPS)-stimulated BMSCs and analyzed their cytotoxicity. We further evaluated the anti-inflammatory and osteogenic ability of the candidate peptide in an *in vitro* inflammatory environment and in an *in vivo* bone defect animal model to provide a basis for their application in jaw bone defect repair.

## 2 Materials and Methods

### 2.1 Polypeptide Screening

#### 2.1.1 Lipopolysaccharide Challenge

Primary BMSCs from 4-week-old male Sprague-Dawley rats were cultured using the whole bone marrow adsorption method. Cells were cultured in alpha-minimal essential medium (HyClone, Logan, UT, United states ), fetal bovine serum (Gibco BRL, Sydney, Australia), and penicillin (Gibco BRL, Gaithersburg, MD, United states ). The cultured BMSCs at passage three were divided into three groups: the control group, 3 μg/ml LPS (*Escherichia coli* LPS; PeproTech, Rocky Hill, NJ, United states ) group, and 5 μg/ml LPS group, and cultured for 24 h.

#### 2.1.2 Peptide Extraction

An appropriate amount of trypsin was added to the cell culture for digestion. Once the cells separated from the Petri dish, 1:1 medium was added to terminate digestion. The sample was centrifuged at 100–200 × *g* for 3–5 min, and the supernatant was discarded. An appropriate amount of phosphate-buffered saline (PBS) was added to the centrifuge tube, and the cell precipitate was blown away using a pasteurization tube. The sample was centrifuged for 3–5 min, and the supernatant was discarded. The centrifuge tube containing the cell precipitate was immediately immersed in liquid nitrogen and stored at −80°C until analysis.

#### 2.1.3 Mass Spectrometry (MS)-Based Identification of Differentially Expressed Polypeptides

The cell samples of the three groups were centrifuged at 1,000 × *g* for 5 min at 4°C, and the supernatant was discarded. Extract (0.4 ml) was added to the cell sample, and the cells were broken using sonication on ice for 10 min. Centrifugation was performed at 2,000 ×*g* at 4°C for 30 min, and the supernatant was collected. After filtering, large molecules such as proteins with molecular weight >10 KDa in the ultrafiltration tube were discarded. Each sample was analyzed using liquid chromatography-MS for 2 h. The eluting solution was water with 0.1% formic acid (FA) and acetonitrile with 0.1% FA. The samples were loaded in a C18 chromatographic column (250 mm × 75 mm, aperture 100 a, size 2 μm) and run at a flow rate of 300 nL/min. Next, the eluting solution was freeze-dried, the acetonitrile was removed, and the solution was stored at −80°C until loading on the mass spectrometer.

MS was performed on a Fusion system (Thermo Fisher). The ion source spray voltage was 2.3 kV, and the fusion mass spectrometer heating capillary was set to 320°C. Data dependence mode was adopted to automatically switch between MS and tandem MS (MS/MS) modes. Full-scan MS uses Orbitrap for level-1 scanning at a mass-charge ratio (*m/*z) of 350–1,600 with the resolution set at 60,000 (*m/z* 200 locations). The maximum ion introduction time was 50 ms, automatic gain control (AGC) was set to 1 × 10^6^, and the parent ions conforming to cascade (MS/MS) fragmentation were fragmented within 2 s using higher energy C-trap dissociation and scanned using Orbitrap at a resolution of 15,000. The scanning range was automatically controlled according to the *m/z* ratio of the parent ions. The minimum scanning range was fixed at *m/z* = 110 and the maximum was 2,000. The minimum ion strength value for MS/MS was set to 50,000, the maximum ion introduction time for MS/MS was set to 100 ms, the AGC was set to 1.0 × 10^5^, and the parent ion selection window was set to 1.6 Dalton. For ions with 1, 2, and three charge numbers, MS/MS collection was conducted, and dynamic exclusion was set as one MS/MS within 10 s for each parent ion, followed by exclusion of 21 s with a collision energy of 30%.

Peptides were identified based on comparison with the MAXQUANT library using the following search parameters: Uniprot Database [*Rattus norvegicus* (Rat), 2020-05-04, Total Entries 36,152, Reviewed entries 8094, Unreviewed entries 28,058], enzyme: unspecific, variable modifiers: Oxidation (M) Acetyl (Protein N—term) fixed to modify, and default parameters for the others. Differentially expressed peptides were identified according to a >2-fold difference, *p* < 0.05, and false discovery rate <1%.

According to the mass spectrum signal intensity, multiple of difference, and structural stability, we finally screened out one peptide (PAP) with the lowest expression under LPS stimulation for further functional verification.

#### 2.1.4 Functional Annotation

Gene Ontology (GO) is an internationally standardized gene function classification system that provides a dynamically updated standard controlled vocabulary to comprehensively describe the properties of genes and gene products in organisms. There are three ontologies in GO that respectively describe the biological processes, molecular function, and cellular component for a gene. The corresponding number of differential proteins was counted for the items annotated using GO.

### 2.2 Cytotoxicity Test

BMSCs were inoculated on 96-well plates, and the cells were treated with different concentrations of PAP for 1,4 and 7days. The Cell Counting Kit-8 (CCK-8; Dojindo Molecular Technology, Japan) assay was used to determine the effect of PAP on BMSC viability according to the manufacturer’s instructions.

### 2.3 ALP Staining of Bone Marrow-Derived Mesenchymal Cells

BMSCs were seeded on control group and PAP group surfaces and incubated for 7 days. At each time point, the samples were washed with PBS carefully, followed by 4% paraformaldehyde fixation for 20 min, and stained using the BCIP/NBT Alkaline Phosphatase Color Development Kit (Beyotime, China) according to the manufacturer’s protocol. The samples were then observed under a stereomicroscope (SZ61, Olympus, Japan). For quantitative evaluation of ALP activity, the samples were rinsed with PBS, lysed with 1% Triton X-100 for 30 min, and activity was detected with the Alkaline Phosphatase Assay Kit (Beyotime, China) according to the kit instructions.

### 2.4 Reverse Transcription-Quantitative Polymerase Chain Reaction (RT-qPCR) Analysis of Bone Marrow-Derived Mesenchymal Cells.

After incubation for 7 days, RT-qPCR was performed to quantitatively assess the expression levels of osteogenic genes (*Bmp2*, *Ocn*, and *Runx2*) in BMSCs. Total RNA was extracted using Axygen RNA Miniprep Kit (Axygen, Union City, CA, United states ) according to the manufacturers instructions. Reverse transcription was completed using Prime Script RT Reagent Kit (Takara Biotechnology, Otsu, Shiga, Japan). qPCR was then performed on ABI 7500 Sequencing Detection System (Applied Biosystems, Foster City, CA, United states ) using SYBR^®^ Premix Ex Taq™ II (Takara Biotechnology). Primers are listed in Table 1.

**TABLE 1:** Sequences of the RT-qPCR.

Gene Name	Forward	Reverse
TNF-α	GGCGGTGCCTATGTCTCA	GGCAGCCTTGTCCCTTGA
IL-1β	AGC​TTC​AGG​CAG​GCA​GTA​TCA​C	CCA​GCA​GGT​TAT​CAT​CAT​CAT​CC
IL-6	TGCCTTCTTGGGACTGAT	CTG​GCT​TTG​TCT​TTC​TTG​TT
iNOS	ATG​GAA​CAG​TAT​AAG​GCA​AAC​ACC	GTT​TCT​GGT​CGA​TGT​CAT​GAG​CAA​AGG
OCN	TCA​CTT​CCG​CCC​GGA​ACC​CT	TGT​CCT​GCC​GGC​CCA​AGA​GA
RUNX2	ATC​ATT​CAG​TGA​CAC​CAC​CA	GTA​GGG​GCT​AAA​GGC​AAA​AG
BMP2	GCA​TGT​TTG​GCC​TGA​AGC​AG	CGA​TGG​CTT​CTT​CGT​GAT​GG

### 2.5 Validation of Anti-inflammatory Effect of PAP

#### 2.5.1 Analysis of Macrophage Polarization Induced by PAP

RAW 264.7 macrophages were seeded in Petri dishes at a density of 2 × 10^5^ cells/mL. After cell adherence, the macrophages were stimulated for 24 h with 100 ng/ml LPS to polarize into the M1 phenotype. Subsequently, M1 macrophages were treated with different concentrations of PAP for 24 h, and the expression levels of M1-related genes encoding TNF-α, IL-1β, IL-6, and induced nitric oxide synthase (iNOS) were detected using RT-qPCR (Table 1).

#### 2.5.2 Intracellular Reactive Oxygen Species (ROS) Detection

Intracellular ROS were detected using a dichloro-dihydro-fluorescein diacetate (DCFH-DA) probe (1:1,000; S0033S; Beyotime Biotechnology, China). In brief, RAW 264.7 macrophages were plated on confocal microscopy dishes (Cellvis, CA, United states ) and cultured with PBS, LPS (100 ng/ml), or LPS (100 ng/ml) and PAP (10 or 50 μg/ml) for 24 h. The cells were then incubated with serum-free culture medium containing 10 μM DCFH-DA for 20 min at 37°C. Next, the cells were stained with Hoechst 33,342 for nuclear fluorescence and washed three times with warm PBS to remove excess dye. The fluorescent images were captured using confocal laser-scanning microscopy (CLSM).

#### 2.5.3 Intracellular Superoxide Detection

Intracellular superoxide was detected using dihydroethidium (DHE) (HY-D0079; Med Chem Express, China). In brief, RAW 264.7 macrophages were co-cultured with 5 μM DHE for 30 min at 37°C after 24 h of incubation with PBS, LPS, and/or PAP as described earlier. Nuclei were stained with Hoechst 33,342, and the cells were washed three times with warm PBS to remove excess dye and observed using CLSM.

#### 2.5.4 Inflammatory Bone Destruction Model Establishment and PAP Treatment

Six-week-old male C57BL/6 mice were obtained from Shanghai West Poole-Baykay Laboratory Animal Co., Ltd (Shanghai, China). This study was approved by the Ethics Committee of Shanghai Tongren Hospital (2021-038). The mice were randomly divided into three groups (*n* = 3 per group): control mice injected with PBS only (300 μL), mice injected with LPS (200 μL) and PBS (100 μL), and mice injected with LPS (200 μL) and 10 μg/ml PAP (100 μL). LPS was dissolved in PBS at a concentration of 1 g/L. In all cases, injections were administered between the subcutaneous tissue and the bone periosteum of the head. The mice were injected three times per week and euthanized 2 weeks later. To reduce pain for euthanasia, the mice were briefly anesthetized in an isoflurane-filled box to induce early unconsciousness and then killed by cervical dislocation.

#### 2.5.5 Micro-computed Tomography (Micro-CT)

The cranium of each mouse was harvested, and the soft tissue around the bone was separated for micro-CT using a μCT-100 scanner (Scanco Medical AG, Brüttisellen, Switzerland). The scanning parameters were 70 kV, 114 mA, and a scanning thickness of 50 μm. Bone mass analysis was performed to evaluate bone damage quantitatively after three-dimensional reconstruction.

#### 2.5.6 Histology and Immunohistochemistry

The calvarial bone tissue of each mouse was fixed in 4% paraformaldehyde for 24 h and then washed overnight. Subsequently, the tissue was decalcified with 10% ethylenediaminetetraacetic acid at 4°C, dehydrated using a graded series of ethanol, and embedded in paraffin after *n*-butyl alcohol exchanges. Each specimen was sectioned to a near-far-median sagittal section thickness of 4 μm. Following sectioning, hematoxylin and eosin (H&E) staining was performed to visualize cranial cap bone defects under a microscope.

After calvarial bone specimens were deparaffinized with dimethylbenzene and processed with a graded series of ethanol concentrations, the TRAP stain was prepared and incubated with the tissues at 37°C for 1 h. Subsequently, the tissues were re-stained with hematoxylin for 2 min, cleared using xylene, and mounted and sealed using neutral gum. The tissue slides were observed under an optical microscope, and TRAP-positive cells (brown) were counted.

Conventional dewaxing treatment for obtaining paraffin sections was performed for immunohistochemistry. The numbers of TNF-α- and IL-1β-positive cells were determined under a microscope.

### 2.6 Statistical Analysis

Multiple Array Viewer was used for hierarchical clustering and visualization of quantitative data samples and gene dimensions. All data were analyzed using SPSS 23.0 software (IBM, Armonk, NY, United states ). The data are expressed as mean ± standard deviation. Data were compared using analysis of variance and t-tests, as appropriate. The level of statistical significance was set at *p* < 0.05*.*

## 3 Results

### 3.1 Polypeptide Screening

**FIGURE 1:**
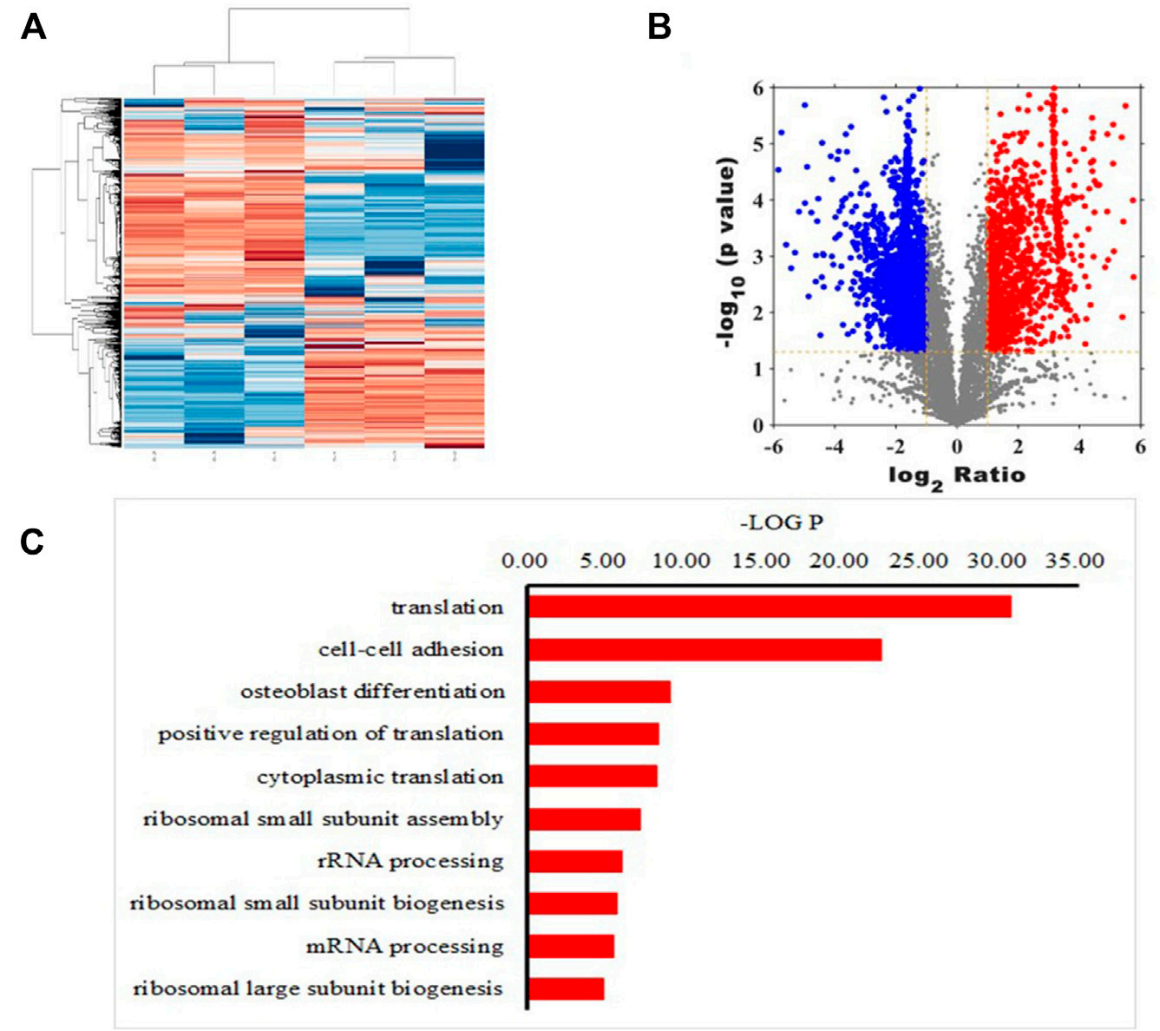
Quantitative peptide analysis. Heat map **(A)** and volcano plot **(B)** showing differences in abundance of peptides screened between lipopolysaccharide (LPS)-treated and control groups. *X*-axis represents the intensity ratio of peptides in the two groups of samples, *Y*-axis represents the *p* value representing inter-group repeatability, and each point in the plot represents an individual peptide. Red and green areas indicate upregulated and downregulated peptides, respectively **(C)** Ten biological processes with the highest protein enrichment corresponding to differential polypeptides.

Mass spectrometry analysis of peptides resulted in heat maps (Figure 1A) and volcano maps (Figure 1B), which show the difference in peptide abundance between the lipopolysaccharide (LPS) treated and control groups We can clearly see the similarity clustering relationship between samples, the similarity clustering relationship between genes, and the distribution of up-regulated and down-regulated polypeptides. In addition, the 10 biological processes with the highest protein enrichment corresponding to differential polypeptides are also reflected in Figure 1C, among which the top five pathways with significant enrichment are translation, cell-cell adhesion, osteoblast differentiation, positive regulation of translation, cytoplasmic translation.

In addition, MS-based polypeptide omics identified a total of 1,538 polypeptides with reduced expression in the LPS-treated rat BMSCs compared with those in the control group, The research arranged all peptides in ascending order according to their expression level in the control group. The top five peptides with significantly decreased expression were PAP, VLVGDGGTGKTT, KKGAKLTPEEEEILNKK, TEDKADVQS and VIYTRNTKGGDAPAAGEDA, then identified PAP as the peptide with the lowest expression in the inflammatory environment. Thus, the research predicted that the addition of PAP to a bone graft material could potentially inhibit further progress of an inflammatory response.

### 3.2 Cytotoxicity of PAP

**FIGURE 2:**
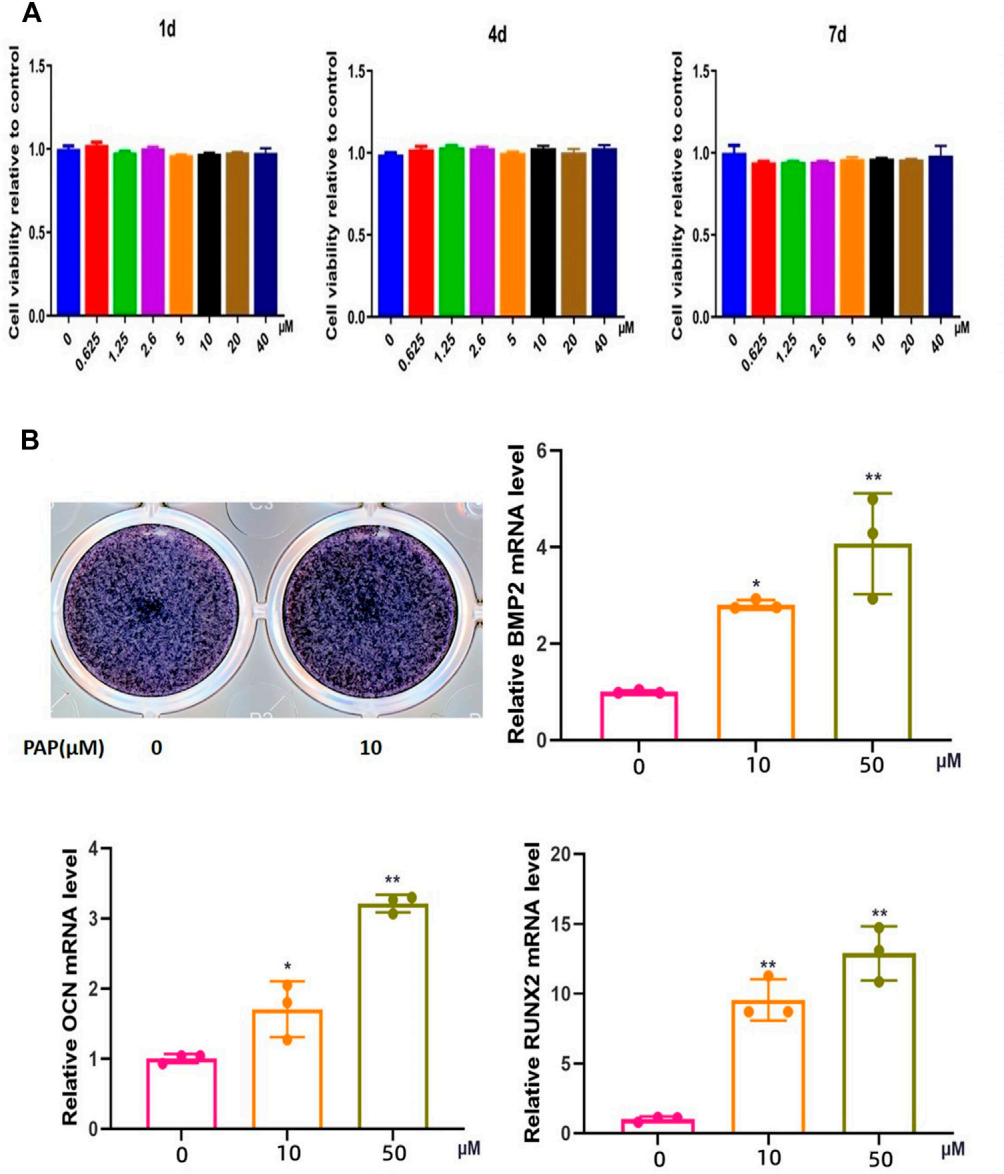
Cytotoxicity of and osteogenesis promotion by polypeptide PAP. CCK-8 assay results on **(A)** 1,4, and 7 d of exposure of rat bone marrow-derived mesenchymal stem cells (BMSCs) to lipopolysaccharide (LPS) and/or PAP **(B)** ALP staining and RT-qPCR of osteogenesis-related genes in rat BMSCs. **p < 0.01, *p < 0.05.

CCK-8 of 1, 4d, 4d and 7 d assay showed that the viability of the BMSCs was not significantly reduced with increase in PAP concentration, indicating that PAP was not significantly cytotoxic to rat BMSCs (Figure 2A).

### 3.3 Osteogenic Potential of PAP in BMSCs

In ALP staining, the positivity rate in the group treated with 10 μM PAP was significantly higher than that in the control group, and RT-qPCR showed that the mRNA expression levels of *Ocn, Bmp2*, and *Runx2* were significantly higher in the PAP-treated cells than in the control group. These levels also gradually increased with increase in PAP concentration, which confirmed that PAP could promote osteogenic induction of rat BMSCs (Figure 2B).

### 3.4 Macrophage Polarization Induced by PAP

**FIGURE 3:**
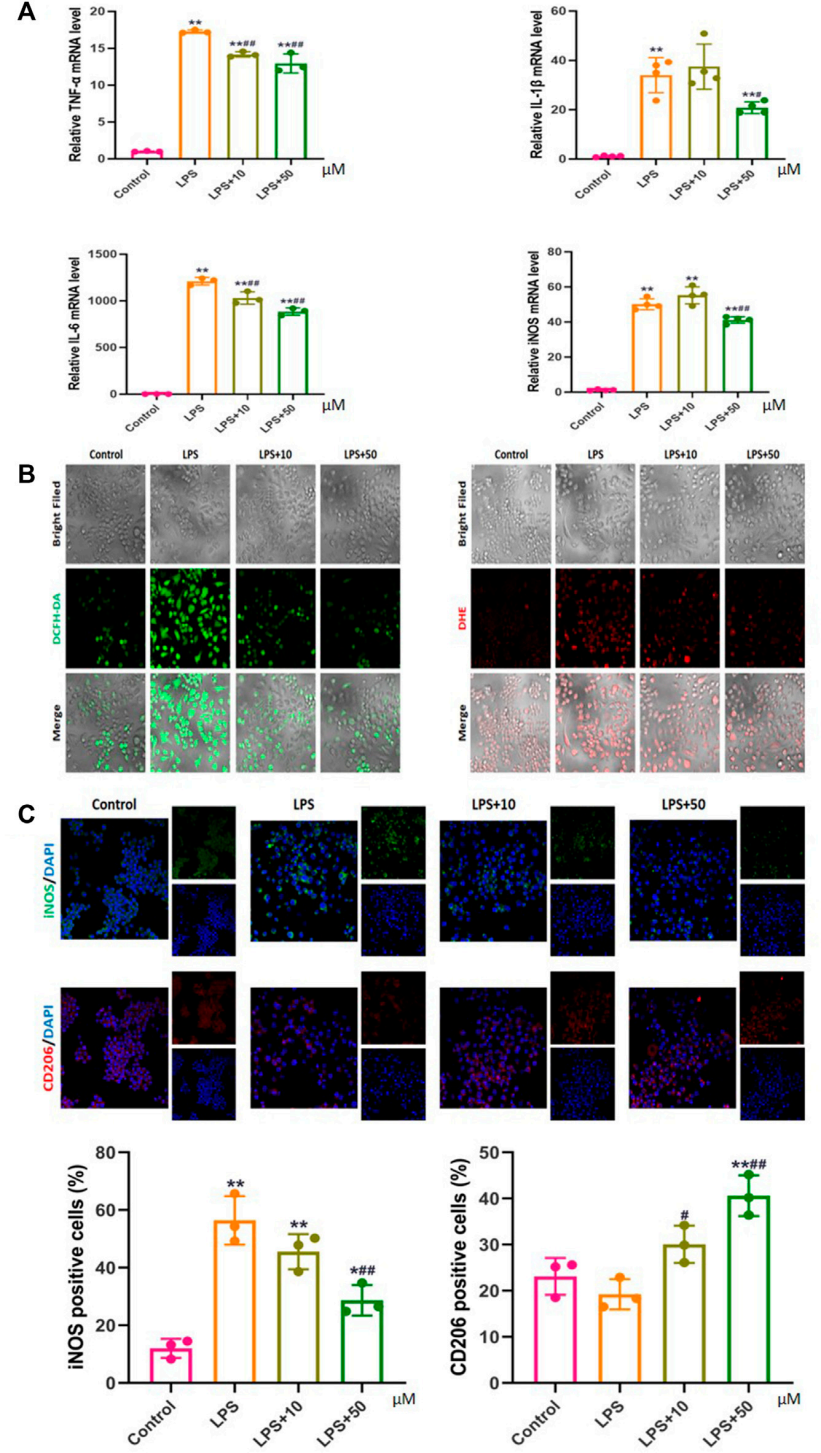
Effect of PAP on inflammation and oxidative stress **(A)** Real-time PCR of pro-inflammatory genes **(B)** Detection of reactive oxygen species (ROS) using DCFH-DA and DHE probes **(C)** Immunofluorescence to detect markers of macrophage polarization. *p < 0.05, **p < 0.01 compared with the control group; ^#^ *p* < 0.05, ^##^ *p* < 0.01 compared with the LPS group.

Compared with those in the control group, the mRNA expression levels of the inflammatory factors TNF-α, IL-1β, IL-6, and iNOS in the LPS-treated RAW 264.7 macrophages were significantly increased, which decreased with the addition of PAP. Among them, the expression levels of TNF-α and IL-6 were dependent on the PAP concentration, with a greater decrease upon addition of a higher concentration of PAP (Figure 3A).

### 3.5 Effect of PAP on Inflammation-Induced Oxidative Stress

PAP treatment significantly inhibited ROS levels in LPS-stimulated inflammatory macrophages, and the inhibition effect was more obvious under high-concentration treatment (Figure 3B). Concurrently, immunofluorescence confirmed that PAP could effectively regulate the polarization behavior of LPS-stimulated macrophages by inhibiting the expression of iNOS on the surface of M1 macrophages and promoting the expression of CD206 on the surface of M2 macrophages in a concentration-dependent manner. These results suggested that PAP has an ideal anti-inflammatory and antioxidant effect on inflammatory macrophages (Figure 3C).

### 3.6 Micro-CT Results

**FIGURE 4:**
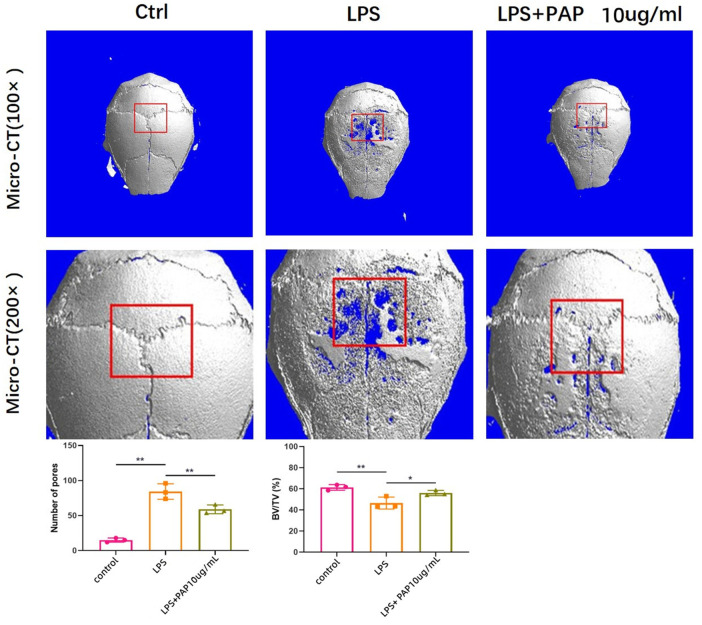
Micro computed tomography of mouse cranial bone resorption. Red box area represents bone resorption lacunae, quantitatively analyzed to measure new bone volume percentage. **p < 0.01, *p < 0.05.

The formation of cranial resorption pits *in vivo* was assessed using micro-CT. The formation of cranial resorption pits was more apparent in the LPS-injected group than in the PBS-only control group. However, the number of LPS-induced bone resorption pits was significantly reduced in mice treated with PAP compared with that in the LPS-treated group. Statistical analysis of the micro-CT scans showed that the skull bone mass of mice in the LPS group was reduced compared with that of mice in the blank control group. The bone mass of the LPS + PAP group was higher than that of the LPS group. Significant differences were found between the PAP group and the PBS or LPS groups (Figure 4).

### 3.7 H-E Staining and TRAP Staining of the Calvarial Bone

**FIGURE 5:**
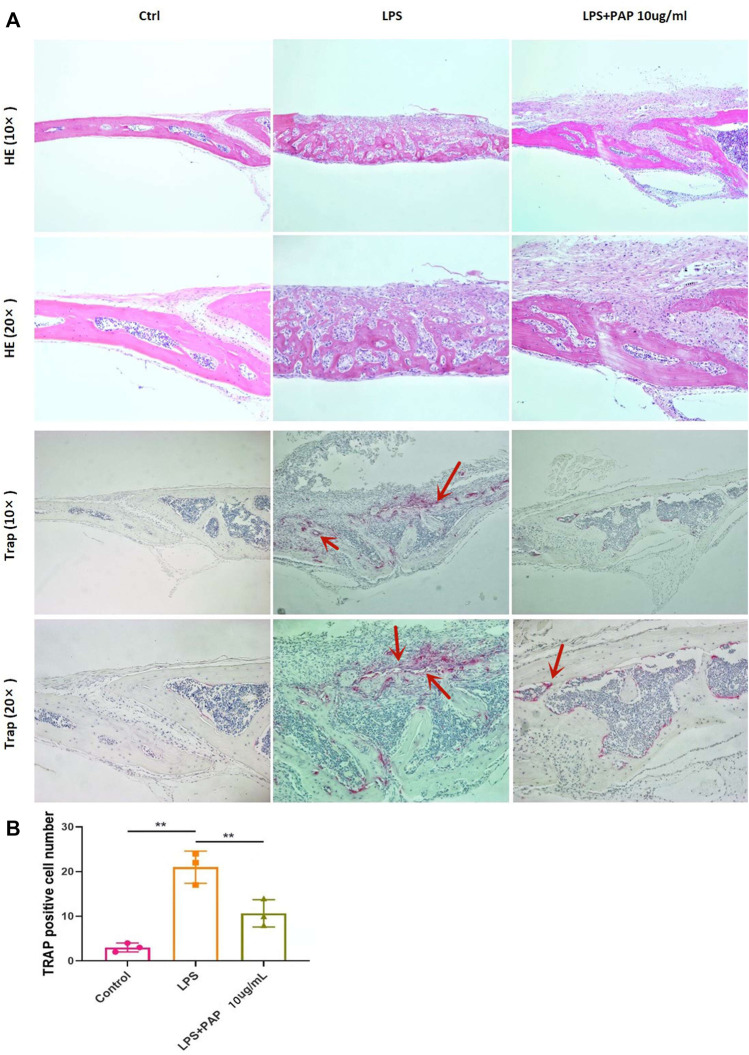
Hematoxylin and eosin (H,E) and TRAP staining of cranial bone absorption **(A)** Representative images of H&E and TRAP staining; red arrow indicates osteoclasts **(B)** Quantitative analysis of TRAP-positive cells. **p < 0.01.

H&E staining showed that the cranium of LPS-treated mice was notably damaged compared with that of mice in the PBS control group. However, in mice treated with PAP, the cranial damage induced by LPS was significantly inhibited, and bone resorption was significantly reduced. TRAP staining showed that PAP inhibited the osteoclastogenesis induced by LPS (Figure 5A). Statistical analysis of TRAP staining showed that the number of TRAP-positive cells (representing osteoclasts) increased in the LPS group compared with that in the control group. Concurrently, the number of osteoclasts in the LPS + PAP group was lower than that in the LPS-only group (Figure 5B).

### 3.8 Immunohistochemical Staining of the Cranial Bone

**FIGURE 6:**
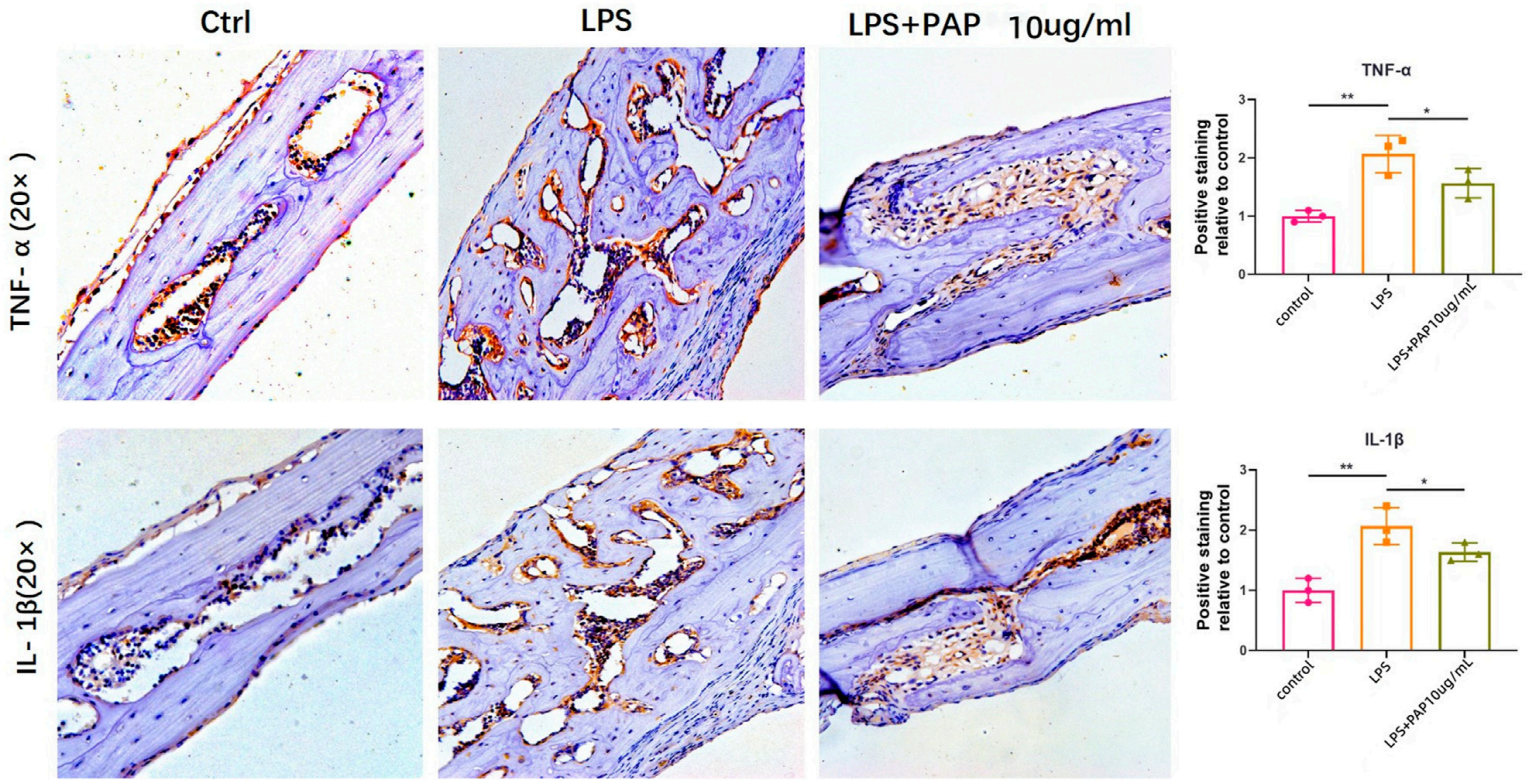
Immunohistochemical staining of inflammatory cytokines. Numbers of TNF-α or IL-1β-positive cells in the cranium of mice treated with lipopolysaccharide (LPS) with or without PAP were quantified. **p < 0.01, *p < 0.05.

Immunohistochemical staining of the cranial bone revealed that TNF-α and IL-1β-positive cells of LPS group was significantly higher compared with that of mice in the PBS control group, but they were significantly lower in the LPS + PAP group than those in the in the LPS group. The number of TNF-α and IL-1β-positive cells was graphed and subjected to statistical analysis. The number of TNF-α and IL-1β-positive cells increased in the LPS group compared to the control group. Concurrently, the number of TNF-α and IL-1β-positive cells in the LPS + PAP group was lower than that in the LPS group alone. Compared to the PAP group, the PBS group and LPS group exhibited statistically significant differences. These results suggest that PAP has an ideal anti-inflammatory and antioxidant effect (Figure 6).

## 4 Discussion

Jaw defects are a common cause of oral and maxillofacial surgery, which affect mastication, swallowing, breathing, and other physiological functions, and thus significantly reduce quality of life. The existing repair methods for jaw defects induce an inflammatory environment, which reduces the osteogenesis efficiency. The process of osteogenesis is mainly influenced by the balance between osteoblast and osteoclast activity. An inflammatory microenvironment subjects BMSCs to ischemia, hypoxia, and inflammation, which has a serious impact on the self-renewal ability and multi-directional differentiation potential of the cells, leading to decrease in their osteogenic differentiation ability and inhibition of the bone formation ability of osteoblasts. The presence of local inflammatory factors further induces signaling pathways related to BMM differentiation and bone resorption function (Mori et al., 2009; Sokos et al., 2015).

Therefore, a bone repair material that can effectively inhibit the inflammatory response would improve the osteogenic efficiency to achieve a desired outcome. In the present study, we innovatively harnessed the properties of polypeptides, which have small molecular weights, readily enter cells, and produce metabolites with no side effects, to address the limited efficiency of traditional bone repair materials. A total of 1,538 differentially expressed peptides were screened out in LPS-stimulated BMSCs with lower expression than that in the control group and further focused on the PAP peptide with the lowest expression under inflammatory conditions overall. *In vitro* assays showed that PAP was non-toxic to cells and could promote the osteogenic induction of rat BMSCs. Moreover, PAP inhibited LPS-induced increase in the expression of inflammatory M1 macrophage-related genes encoding TNF-α, IL-1β, IL-6, and iNOS. Accumulation of pro-inflammatory cytokines induces production of excessive ROS by stimulating the respiratory burst of inflammatory macrophages (El-Benna et al., 2016). Our intracellular superoxide and ROS detection further confirmed that PAP inhibited ROS production in LPS-activated macrophages. Immunofluorescence also demonstrated that PAP increased the expression of iNOS, an M1 polarization marker, and reduced the expression of CD206, an M2 polarization marker, in a dose-dependent manner. Taken together, the *in vitro* observations indicated that PAP inhibited the inflammatory response, repolarized macrophages from the inflammatory to non-inflammatory phenotype, and suppressed oxidative stress in the inflammatory environment, demonstrating its potential to improve osteogenic behavior in bone repair.

These effects *in vitro* were further confirmed *in vivo* using a mouse skull inflammation model. Micro-CT showed that with increase in PAP concentration, the extent and degree of the skull defect decreased correspondingly. H&E and TRAP staining further showed a reduction in bone destruction with PAP treatment. Finally, immunohistochemistry demonstrated decreases in the numbers of TNF-α- and IL-1β-positive cells with the addition of PAP compared with those in the LPS group. Therefore, our results suggest that PAP is non-cytotoxic and inhibits inflammatory processes, with great potential to improve osteogenic behavior in an inflammatory environment.

## 5 Conclusion

The polypeptide PAP screened from LPS-stimulated BMSCs is not cytotoxic and can inhibit the inflammatory reaction process to promote the smooth progress of osteogenesis. PAP has good osteogenic potential and shows promise as a bone-promoting material. This study thus provides a basis for further study of the osteogenic ability of PAP in the inflammatory environment and its application in the field of jaw defect repair.

However, since this was a preliminary study focused on its anti-inflammatory effects, we did not directly verify the ability of PAP to promote osteogenesis; thus, further *in vivo* and *in vitro* studies using PAP-loaded bone filling materials are needed for validation and to select the most appropriate materials with the ultimate goal of clinical translation.

## Data Availability

The original contributions presented in the study are included in the article/Supplementary Material, further inquiries can be directed to the corresponding authors.
